# Biomimetic cultivation of atrial tissue slices as novel platform for in-vitro atrial arrhythmia studies

**DOI:** 10.1038/s41598-023-30688-8

**Published:** 2023-03-04

**Authors:** Jorik H. Amesz, Natasja M. S. de Groot, Sanne J. J. Langmuur, Hamid el Azzouzi, Vera P. C. Tiggeloven, Manuela M. M. M. van Rooij, P. Knops, Ad J. J. C. Bogers, Yannick J. H. J. Taverne

**Affiliations:** 1 Translational Cardiothoracic Surgery Research Lab, Department of Cardiothoracic Surgery, Erasmus University Medical Center, Rotterdam, the Netherlands Dr. Molewaterplein 40, 3015GD; 2 Translational Electrophysiology, Department of Cardiology, Erasmus University Medical Center, Rotterdam, the Netherlands; 3 Department of Molecular Genetics, Erasmus University Medical Center, Rotterdam, the Netherlands

**Keywords:** Experimental models of disease, Cardiology, Translational research

## Abstract

Living myocardial slices (LMS) are beating sections of intact human myocardium that maintain 3D microarchitecture and multicellularity, thereby overcoming most limitations of conventional myocardial cell cultures. We introduce a novel method to produce LMS from human atria and apply pacing modalities to bridge the gap between in-vitro and in-vivo atrial arrhythmia studies. Human atrial biopsies from 15 patients undergoing cardiac surgery were dissected to tissue blocks of ~ 1 cm^2^ and cut to 300 µm thin LMS with a precision-cutting vibratome. LMS were placed in a biomimetic cultivation chamber, filled with standard cell culture medium, under diastolic preload (1 mN) and continuous electrical stimulation (1000 ms cycle length (CL)), resulting in 68 beating LMS. Atrial LMS refractory period was determined at 192 ± 26 ms. Fixed rate pacing with a CL of 333 ms was applied as atrial tachyarrhythmia (AT) model. This novel state-of-the-art platform for AT research can be used to investigate arrhythmia mechanisms and test novel therapies.

## Introduction

Living myocardial slices (LMS) are ultra-thin sections of intact human myocardium, which are presented as a novel model for translational research^1,2^. LMS overcome most limitations of conventional myocardial cell cultures and allow for a high degree of in-vivo representativeness, due to the maintained 3D architecture and intact cell–cell interactions of human cardiac biopsies^1^. The tissue slices remain beating in culture for several weeks in a biomimetic system with near-physiological preload and continuous electrical stimulation^3,4^.

To date, LMS have been mainly produced from ventricular biopsies^5,6^ and only few studies reported on slice production from atrial biopsies^7–10^. However, lack of a biomimetic cultivation system resulted in rapid degeneration of these atrial slices under unloaded culture conditions. Moreover, the production of atrial LMS is complex due to the variable thickness of the atrial wall caused by the pectinate muscles^11^.

In this paper, we present an innovative method to reliably produce and cultivate atrial myocardial tissue slices which can be used as a platform for in-vitro atrial arrhythmia studies by applying different pacing rates. As such, atrial LMS can further unravel the underlying mechanisms of atrial arrhythmias, including atrial fibrillation (AF), and bridge the gap between in-vivo and in-vitro studies.

## Results

**Figure 1:**
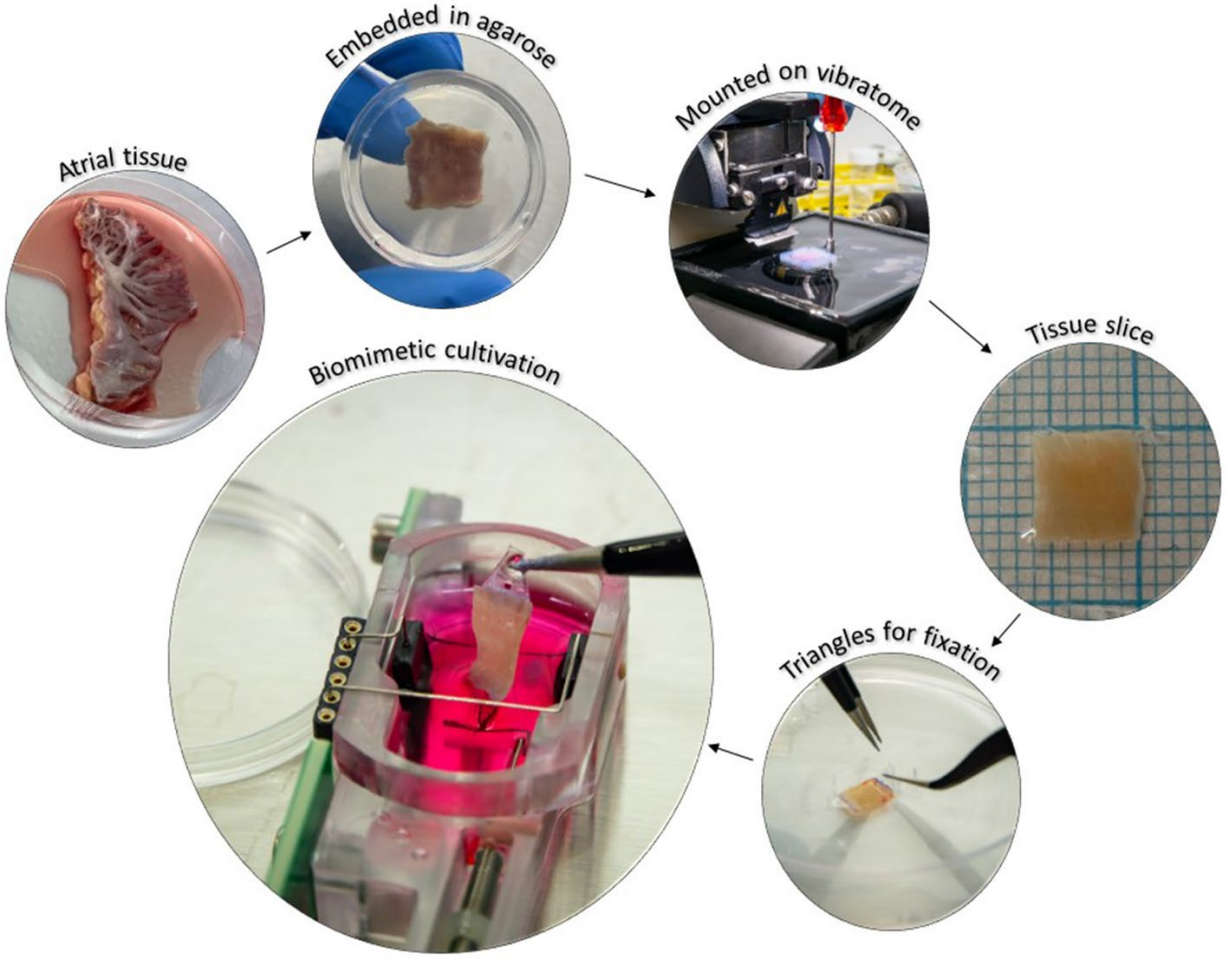
Schematic overview of atrial tissue slice production.

**Figure 2:**
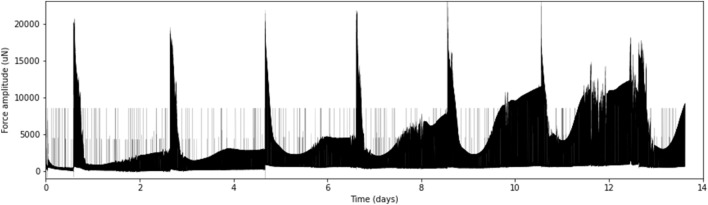
Exemplary contractility traces of an atrial tissue slice for 14 days in cultivation. Culture medium was refreshed every two days with subsequent isoprenaline administration, characterized by short-term increases in twitch force amplitude. Experimentation was stopped on day 14 of biomimetic cultivation.

We produced 68 LMS consisting of 23 left atrial and 45 right atrial tissue slices from 15 patients undergoing cardiac surgery (median age 60 years, 73% male, 47% with AF history) (Table 1). This was achieved by modification of the original protocol designed for ventricular biopsies with adjusted vibratome settings (advance speed 0.02 mm/s, vibration amplitude 2.0 mm), isoprenaline enhancement (1 µM) and shortened stimulation cycle length (CL) (1000 ms) (Fig. 1). Application of near-physiological preload (~ 1 mN) and electrical field stimulation (50 mA stimulation current and 3 ms pulse charge duration) resulted in a beating atrial LMS culture for 1 to 2 weeks (Supplementary Video 1), after which cultivation was stopped (Fig. 2). Some LMS presented with spontaneous contractions prior to initiation of electrical stimulation, suggesting automaticity or triggered activity of atrial cells. Isoprenaline enhancement (1 µM) was needed to maintain sufficient contractions in the LMS.

**Table 1:** Baseline characteristics of patients who donated biopsies for LMS production. Patients were classified in AF classes based on information from their medical records.

	Age (years)	Sex	AF class	RA LMS	LA LMS
1	69	Male	No AF	5	
2	67	Female	Persistent		1
3	72	Female	Persistent		3
4	0.4	Female	No AF	3	2
5	77	Male	Persistent		5
6	74	Male	Persistent		3
7	12	Female	No AF	1	
8	64	Male	No AF	3	
9	46	Male	Persistent	5	
10	59	Male	Paroxysmal	4	4
11	63	Male	No AF	7	
12	60	Male	No AF		5^†^
13	60	Male	No AF	2	
14	47	Male	Paroxysmal	7	
15	22	Male	No AF	8	
**Total**	**Median: 60**	**% Male: 73**	**% AF: 47**	**45**	**23**

*AF* atrial fibrillation, *LA* left atrium, *LMS* living myocardial slices. *RA* right atrium.

^†^ Pulmonary vein antral slices.

**Figure 3:**
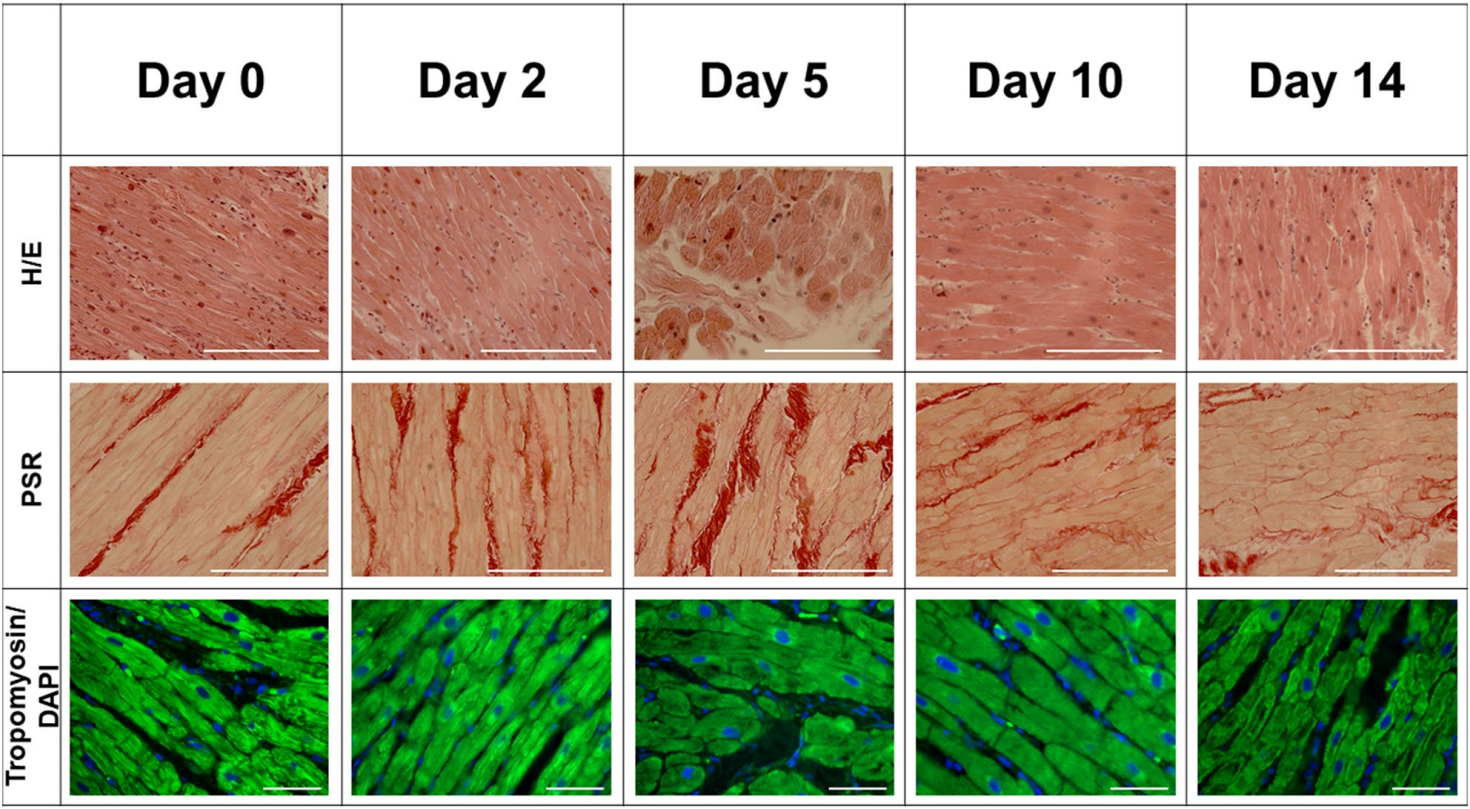
Microscopical images of LMS during various stages of two-week cultivation. LMS derived from the same sample obtained from the same patient, yet different layers of the atrial wall. Scale bar = 100 µm.

An important aspect of LMS is their multicellularity with retained cardiac cellular composition, architecture and physiology of the heart. Figure 3 shows microscopical images of LMS from the right atrium of one patient (male, 22 y/o) during various stages of cultivation. Haematoxylin and Eosin (H&E) stained sections of LMS of day 0 to day 14 were examined under light microscopy and showed no signs of cardiomyocyte disarray and preservation of unidirectional fibre orientation throughout the cultivation period (Fig. 3). Cardiac fibroblasts maintained physiological interactions with the other cardiac cells and the extracellular matrix (ECM), as shown with Picrosirius red (PSR) staining. These images showed the presence of cardiac fibroblasts and intermingling of fibrotic tissue and cardiomyocytes before start of LMS cultivation (Day 0) without increase in the amount of fibrosis during cultivation of LMS up to day 14 (Fig. 3).

Cardiac muscle contraction occurs through mutual sliding between the thick and thin filaments by repeated association and dissociation of myosin heads and actin filaments. Tropomyosin is an essential sarcomeric component, stabilising the thin filament and facilitating actin's interaction with myosin. Our LMS slides showed stable expression of Tropomyosin throughout the 14 days of cultivation indicating a consistent viable and contractile phenotype of the LMS (Fig. 3).

Programmed electrical stimulation with decremental CL between standard and extra stimuli (960 to 140 ms) was used to determine the refractory period in 32 LMS from 6 patients (Supplementary Fig. 1) and was 192 ± 26 ms. Subgroup analysis showed that the mean refractory period was 202 ± 29 (range: 140–260) ms in patients with AF (*n* = 3) and varied more between LMS from the same patient, compared to patients without AF (178 ± 10 (range: 160–200) ms, *n* = 3). Yet, no statistical testing was performed between groups due to low sample size and different clinical presentations of AF.

**Figure 4:**
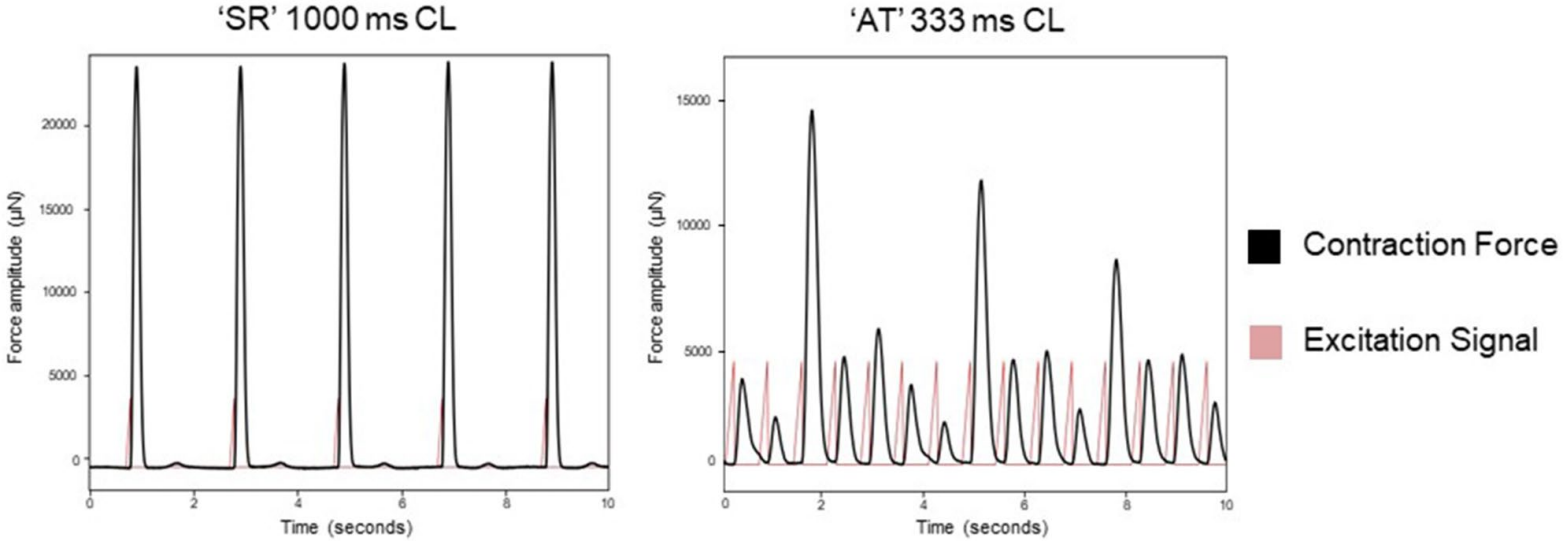
Atrial tachyarrhythmia (AT) model with stimulation at 333 ms CL versus 1000 ms CL as model for sinus rhythm (SR). Contraction force was decreased during tachypacing and there appeared to be more beat-to-beat variation.

Tachypacing with a CL of 333 ms was applied as atrial tachyarrhythmia (AT) model to the same 32 LMS, of which 4 slices were excluded due to a lower contraction force than 300 µN. Contractility characteristics of 1000 ms and 333 ms CL stimulation were compared in the remaining 28 LMS, as model for sinus rhythm (SR) and AT respectively (Table 2). A description of the different contractility parameters can be found in Supplementary Fig. 2. All parameters differed significantly between the SR and AT stimulation frequencies. Contraction force decreased in the AT pacing model and there was a trend towards more beat-to-beat variation during tachypacing, of which an example is presented in Fig. 4.

**Table 2:** Comparison of contractility parameters between sinus rhythm (SR) with 1000 ms CL and atrial tachyarrhythmia (AT) with 333 ms CL stimulation, presented as median (range). F_max_ and A_peak_ were corrected with LMS area.

Parameter	‘SR’ 1000 ms CL median (range) (*n* = 28)	‘AT’ 333 ms CLmedian (range) (*n* = 28)	*p* value
F_max_ (µN/mm^2^)	106.5 (8.9–310.4)	50.2 (4.8–210.6)	< 0.001
A_peak_ (µN.s/mm^2^)	13.1 (1.0–30.9)	5.4 (0.8–21.0)	< 0.001
CD (ms)	198 (140–500)	175 (120–300)	< 0.001
TTP (ms)	90 (60–370)	70 (50–145)	< 0.001
TTR (ms)	110 (70–290)	100 (70–170)	0.009
CD_50_ (ms)	110 (70–290)	90 (60–140)	< 0.001
dF/dt_max_ (mN/s)	43 (4.2–138.7)	28.9 (4.8–118.5)	0.024
dF/dt_min_ (mN/s)	− 39.8 (− 109.4 to − 4.0)	− 21.0 (− 100.9 to − 3.9)	0.007

*A*_peak_ peak area, *CL* cycle length, *CD* contraction duration, *CD*_50_ width at 50% of peak height, *dF/dt*_max_ steepest positive slope, *dF/dt*_min_ steepest negative slope, *F*_max_ maximum contraction force, *TTP* time to peak, *TTR* time to relaxation.

Sub-analyses of patients with and without AF were presented in Supplementary Tables 1 and 2. No statistical testing was performed between patient groups due to a low sample size of *n* = 3 in both groups and different clinical presentations of AF. LMS from patients with and without history of AF showed a similar decline in contractile force during tachypacing, which is why LMS were pooled for all further analyses (Supplementary Tables 1 and 2).

## Discussion

To our knowledge this is the first paper that presents a method to reliably produce human atrial LMS and cultivate them for 1 to 2 weeks in a near-physiological state using a biomimetic system. This model shows high in-vivo representativeness, due to the use of human specimen which maintain 3D architecture with intact cellular interconnections and cell–cell communication. Moreover, LMS from patients with arrhythmia enable the opportunity to directly study the effect of atrial arrhythmias, including AF, on structural and electrical remodeling in a petri-dish^2^.

To date, LMS production of atrial biopsies was limited due to the fact that its production from the thin atrial wall is technically challenging and atrial LMS tend to rupture more easily. Adjustments to the ventricular LMS protocol were needed in order to produce intact atrial LMS. The advance speed of the vibratome was decreased and vibration amplitude increased (0.02 mm/s, 2.0 mm), in comparison to the presented settings for ventricular slices (0.07 mm/s, 1.3 mm)^3^. These adjusted settings for atrial specimen resulted in 68 viable atrial LMS.

A stimulation CL of 1000 ms was chosen for cultivation, as compared to Fischer et al. who maintained a CL of 2000 ms for the cultivation of ventricular LMS^3^. This alternate approach could be explained by differences in cardiac action potentials of atrial and ventricular cardiomyocytes. Atrial cardiomyocytes action potentials display a shorter phase 2, resulting in decreased inward calcium currents^12,13^. Subsequently, this also results in a decreased calcium-induced-calcium-release (CICR) by the sarcoplasmic reticulum, which is essential for cardiac contraction. When the stimulation frequency increases, more calcium accumulates in the cardiomyocytes, since the outward calcium channels cannot cope with the increased amount of calcium ions entering the cell per minute, resulting in improved myocardial tension, also known as the Bowditch effect^14^.

In order to prevent fading of contractions over time, we observed that atrial LMS needed stimulation with isoprenaline. Stimulation of β-adrenoreceptors with isoprenaline results in the production of cAMP, indirectly leading to higher intracellular calcium concentrations^15^. The need for this inotropic stimulation of atrial LMS, could again be explained by the shorter phase 2 of the atrial cardiac action potential, or by atrial myocardium generating less active tension in general^16^, due to atrial myocytes expressing a different form of myosin light chains (MLC)^17^.

Histology of LMS showed clear unidirectional fibre orientation, indicating good handling of the vibratome during LMS production and preservation of the native preferred fibre orientation of the cardiomyocytes in-vivo. Nuclei of cardiac fibroblasts were observed in between cardiomyocytes, demonstrating a co-culture of multiple cell-types in LMS, in accordance with work on ventricular LMS^18,19^. The amount of fibrosis did not seem to increase during culture, suggesting near-physiological cultivation conditions without excessive cardiac injury. Yet, no quantitative analysis on the amount of fibrosis was performed.

After validation of the method to produce and cultivate atrial LMS for the first 8 patients, decremental pacing to determine the refractory period was applied to LMS of the ensuing 6 patients,. The refractory period was determined at 192 ± 26 ms, which is line with the atrial refractory period in-vivo^20^. LMS from patients with AF showed more inter-slice variability within the same biopsies, compared to patients without AF. This observation suggests a higher degree of intramural dispersion in refractoriness of the atrial wall in patients with AF. Dispersion of refractoriness is an accepted mechanism of cardiac tachyarrhythmias, but has so far only been investigated in the intact human heart on either the endo- or epicardium^21^. Yet, LMS enable the opportunity to investigate various intramural layers and not only the endo- or epicardium^22^. As the sample size of the LMS was too small, we did not take transmural variability into account and further studies are needed to investigate these initial observations.

Chronic tachypacing with 333 ms CL was applied as model of AT. Tachypacing to mimic atrial arrhythmias has already been established in various other cellular and animal models^23^ and unloaded LMS^8–10^. We observed a lower median contraction force in the LMS during tachypacing. This also occurs during arrhythmia episodes such as AF in human^24^, therefore suggesting that our tachypacing of LMS mirrors contractile remodeling observed in-vivo. This is in accordance with previous studies using atrial LMS in which tachypacing was applied as model for AT/AF and showed AF-associated remodeling responses on a biochemical and cellular level^8,9^.

Automaticity was observed in some atrial LMS, suggesting the presence of pacemaker activity in the cardiomyocytes of these slices, also observed by Kang et al.^7^. Remarkably, we also observed this automaticity in LMS produced from left atrial biopsies, indicating the presence of secondary or pathological pacemaker cells. This creates opportunities to produce LMS of ectopic pacemaker sites of the atria, which play an important role in the initiation and pathogenesis of AF and study their structural and electrical behavior on a micro-scale^7,25^.

In this report, we described a method to reliably cultivate atrial LMS and use it as tachypacing model for atrial arrhythmias. For future research, programmed electrical stimulation can e.g. be performed intermittently to mimic paroxysmal AF by alternating SR with episodes of AF. We can extend this arrhythmia platform by adding electrophysiological measurements using a micro-electrode mapping array, to examine excitation–contraction coupling of LMS. Beating tissue slices with intact cellular micro-environment will then provide contractile, electrical, biochemical and structural parameters, combined into one single platform^1^. Human atrial LMS provide a realistic pre-clinical platform for screening of novel anti-arrhythmic drugs by simply pipetting compounds to the culture medium. As such, the technology has great potential in revolutionizing the cardiac drug development pipeline, which is currently hampered by high failure rates of clinical trials.

Hence, atrial LMS open the way for a wide variety of atrial arrhythmia experiments, which were previously constrained by limitations in the in-vivo representativeness of cellular and animal models, and therefore difficult to extrapolate to humans. The presented model allows for different programmed electrical stimulation protocols to be applied to mimic atrial arrhythmias such as AF, and serves as a novel state-of-the-art platform for arrhythmia research. This hopefully leads to breakthroughs in the understanding of atrial arrhythmia and AF mechanisms and bridging of the gap between in-vitro and in-vivo translational studies.

## Methods

Human tissue samples of the left and right atrium were collected from residual material from cardiac surgical procedures, such as atrial amputations and heart transplantations. Research using this material was approved by the medical ethics committee of the Erasmus MC (MEC-2020–0988) and participants were informed on use of their residual material, in accordance with local regulations and guidelines.

### LMS production

After retrieval, biopsies were immediately submerged in 4 °C Tyrode solution in the operation theatre and transported to the lab. Other groups have already presented a standardized slicing protocol for LMS, albeit for ventricular tissue^3,5^. Slicing of atrial tissue was deemed more difficult because of inherent atrial trabecularization leading to a variable wall thickness^11^. Furthermore, adipose depositions are more apparent in atrial tissue in comparison to ventricular biopsies^26,27^. On the other hand, when coarse trabeculations are present, they provide a solid biopsy for LMS with clear longitudinally oriented muscle fibres.

Within 24 h after retrieval, atrial tissue specimen were dissected to tissue blocks of approximately 1 cm^2^, subsequently embedded in 37 °C 4% agarose, and cooled until solidification. The agarose block was mounted, with epicardial surface down, on the stage of a precision-cutting vibratome (VT 1200 S, Leica Biosystems). Tissues were cut in tangential orientation with the blade moving parallel to the fibre direction, and a thickness of 300 µm to ensure oxygen diffusion through the cell layers^5^. Advance speed of the vibratome was set to 0.02 mm/s, since the 0.07 mm/s used for ventricular slices appeared to be too fast for atrial slices^7^. Horizontal vibration amplitude was set to 2 mm, as compared to 1.3 mm for ventricular LMS production. In this manner, 3–7 myocardial slices could usually be produced from atrial biopsies, depending on the thickness of the atrial wall (Fig. 1).

Small custom-made plastic triangles were fixated with histoacryl to both ends of the slices with longitudinally aligned fibre orientation in between^3^. The slices were then transferred to biomimetic cultivation chambers (BMCC) with 2.4 mL 37 °C culture medium (Medium-199 supplemented with penicillin–streptomycin, insulin-transferrin-selenite and 2-mercaptoethanol) and cultured as previously described^3^. In short, BMCCs were placed in a standard 37 °C 5% CO_2_ incubator and placed on a rocking plate for continuous agitation. Preload on the LMS was immediately set to 1 mN. Stimulation was initiated with a CL of 1000 ms (50 mA stimulation current and 3 ms pulse charge duration).

### LMS cultivation

Culture medium was refreshed after 1, 24 and subsequently every 48 h by replacing 1.6 of 2.4 mL in the BMCC. Preload was readjusted to 1 mN at every medium exchange. Isoprenaline was added after each medium exchange, maintaining the final concentration constant at 1 µM. Without isoprenaline enhancement, LMS contraction force quenched to a minimum level. Contraction force was measured using dedicated MyoDish Software^3^.

### Histology

LMS (*n* = 8) from one patient were used for histological analyses at various stages of cultivation (days: 0–2–5–10–14). LMS were washed in phosphate buffered saline (PBS), fixed with 4% paraformaldehyde (PFA), followed by paraffin embedding and sectioning at 7 µm. These sections were cut and mounted on coated slides and dried overnight at 37 ℃. Slides were dewaxed in xylene and hydrated using graded alcohols to tap water. The sections were consequently stained with haematoxylin and eosin (H&E; Sigma) for histological analysis and Sirius Red (Sigma) for detection of fibrillar collagen, and mounted using fluoromount (Southern Biotech). For immunofluorescence, sections were permeabilized with 0.1% Triton X-100 (Sigma), dissolved in 1% BSA in PBS for 10 min and blocked with 10% goat serum in PBS for 60 min. Then, the slides were incubated overnight with primary anti-Tropomyosin (1:250, Sigma T9283) diluted in 0.1% BSA in PBS at 4 °C. Secondary antibody incubation was performed at room temperature for one hour, followed by mounting with VECTASHIELD containing DAPI (Vectorlabs). Images were taken by a blinded investigator using the Zeiss LSM‐870 microscope.

### Atrial LMS refractory period

Refractory period was determined by decremental pacing with a fixed S1-rate of 2000 ms and decreasing the extra S2-stimuli delay from 960 to 140 ms with decrements of 20 ms. The first interval that did not show any capture on the S2-stimulus was defined as the refractory period (Supplementary Fig. 1).

### Atrial tachyarrhythmia model

Stimulation with a CL of 333 ms was applied for three minutes as tachypacing model of AT to assess its acute effects on contractility, compared to the same LMS stimulated at 1000 ms CL as model of SR. Contractility parameters were assessed by averaging 30 s of data before and during tachypacing. For each contraction, force amplitude (F_max_), peak area (A_peak_), peak width at 50% of the maximum amplitude (CD_50_), contraction duration (CD), time to peak (TTP), time to relaxation (TTR), steepest positive slope (dF/dt_max_) and steepest negative slope (dF/dt_min_) were extracted from the system recordings with the peak analysis module of LabChart 8 software (ADInstruments)^28^. The different parameters were defined as shown in Supplementary Fig. 2. Start and end of the peak were chosen at 10% away from the baseline to compensate for baseline noise. LMS with an F_max_ of less than 300 µN at 1000 ms CL stimulation were excluded from analysis. Force amplitude and peak area were adjusted with the mm^2^-area of the tissue slice, since bigger slices are expected to generate more force^4^. Medians of 30 s of contractility data were calculated for each individual LMS and pooled by calculating a median and range of the medians of all LMS. Histograms, QQ-plots and Shapiro–Wilk tests were used to determine if differences between 1000 and 333 ms CL stimulation were normally distributed. In case of normal distribution, a paired t-test was used and a Wilcoxon one-sample test otherwise. A *p* value of ≤ 0.05 was considered statistically significant.

### Informed consent

Research using residual material from open-heart surgeries for the BEAT study (MEC 2020–0988) was approved by the medical ethics committee of the Erasmus MC, Rotterdam, the Netherlands. Patients were informed about use of their surgical residual material for scientific research and samples were used unless patients explicitly objected (opt-out method of consent), in accordance with local regulations and guidelines.

## Supplementary Information

Supplementary Information 1.

Supplementary Video 1.

Supplementary Information 2.

## Data Availability

All data generated and analysed during the current study are available from the corresponding author on reasonable request.
