# Supplementary material for: Biomimetic cultivation of atrial tissue slices as novel platform for in-vitro atrial arrhythmia studies
Source: Sci Rep. 2023 Mar 4;13:3648. doi: 10.1038/s41598-023-30688-8 (PMC9985600; doi:10.1038/s41598-023-30688-8)
Supplement: Supplementary file 1 — Supplementary Information 1. [file 41598_2023_30688_MOESM1_ESM.docx]

**Supplementary materials**

Video

**Supplementary video.** Cardiac contractions of an atrial living myocardial slice with exemplary measurements of contractile force.


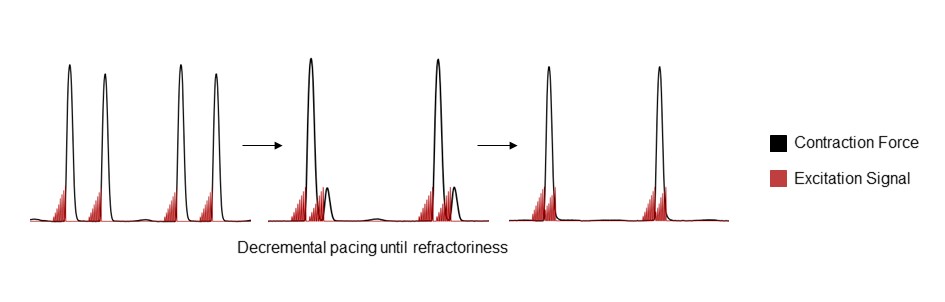


**Supplementary Figure 1.** Refractory period is determined with decremental pacing, defined as the time-delay of the second stimulus which does not lead to capture of contraction**.**


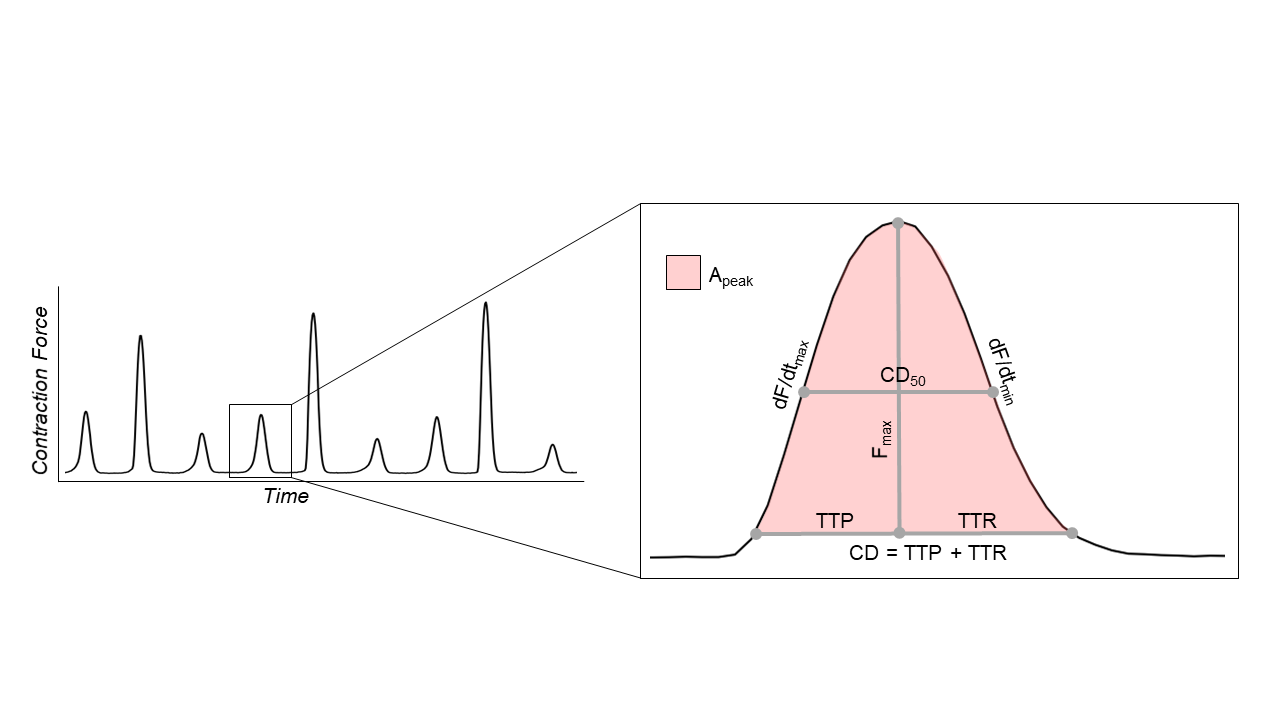

**Supplementary Figure 2.** Definitions of different contractility parameters. A_peak_ = peak area. CD = contraction duration. dF/dt_max_ = steepest positive slope. dF/dt_min_ = steepest negative slope. F_max_ = maximum contraction force. TTP = time to peak. TTR = time to relaxation. CD_50_ = width at 50% of peak height.
