# Supplementary material for: Biomimetic cultivation of atrial tissue slices as novel platform for in-vitro atrial arrhythmia studies
Source: Sci Rep. 2023 Mar 4;13:3648. doi: 10.1038/s41598-023-30688-8 (PMC9985600; doi:10.1038/s41598-023-30688-8)
Supplement: Supplementary file 3 — Supplementary Information 2. [file 41598_2023_30688_MOESM3_ESM.docx]

**Supplementary Tables**

| **Patients with history of AF (*n*=3)** | | |
| --- | --- | --- |
| **Parameter** | **‘SR’ 1000 ms CL**  **median (range) (*n=*16)** | **‘AT’ 333 ms CL**  **median (range) (*n=*16)** |
| **F_max_ (µN/mm^2^)** | 120.6 (18.1 – 310.4) | 63.3 (4.8 – 210.6) |
| **A_peak_ (µN.s/mm^2^)** | 18.5 (1.7 – 30.9) | 7.0 (0.8 – 21.0) |
| **CD (ms)** | 218 (150 – 500) | 190 (140 – 300) |
| **TTP (ms)** | 100 (80 – 370) | 70 (60 – 145) |
| **TTR (ms)** | 110 (70 – 290) | 110 (70 – 130) |
| **CD_50_ (ms)** | 120 (95 – 290) | 90 (80 – 140) |
| **dF/dt_max_ (mN/s)** | 35.6 (7.9 – 126.4) | 22.5 (4.8 – 118.5) |
| **dF/dt_min_ (mN/s)** | -38.0 (-10.9 – -7.6) | -17.3 (-100.9 – -3.9) |

**Supplementary Table 1.** Comparison of contractility parameters in slices obtained from patients with a history of AF between sinus rhythm (SR) with 1000 ms CL and atrial tachyarrhythmia (AT) with 333 ms CL stimulation, presented as median (range). F_max_ and A_peak_ were corrected with LMS area.

A_peak_ = peak area. CL = cycle length. CD = contraction duration. CD_50_ = width at 50% of peak height. dF/dt_max_ = steepest positive slope. dF/dt_min_ = steepest negative slope. F_max_ = maximum contraction force. TTP = time to peak. TTR = time to relaxation.

| **Patients without history of AF (*n*=3)** | | |
| --- | --- | --- |
| **Parameter** | **‘SR’ 1000 ms CL**  **median (range) (*n=*12)** | **‘AT’ 333 ms CL**  **median (range) (*n=*12)** |
| **F_max_ (µN/mm^2^)** | 90.4 (8.9 – 176.9) | 40.1 (11.9 – 74.6) |
| **A_peak_ (µN.s/mm^2^)** | 8.7 (1.0 – 16.2) | 3.1 (1.1 – 6.7) |
| **CD (ms)** | 190 (140 – 220) | 165 (120 – 220) |
| **TTP (ms)** | 90 (60 – 100) | 70 (50 – 80) |
| **TTR (ms)** | 110 (80 – 120) | 95 (75 – 170) |
| **CD_50_ (ms)** | 105 (70 – 120) | 90 (60 – 100) |
| **dF/dt_max_ (mN/s)** | 60.5 (4.3 – 138.7) | 34.2 (8.4 – 67.4) |
| **dF/dt_min_ (mN/s)** | -44.5 (-99.0 - -4.0) | -25.0 (-54.7 - -6.5) |

**Supplementary Table 2.** Comparison of contractility parameters in slices from patients without a history of AF between sinus rhythm (SR) with 1000 ms CL and atrial tachyarrhythmia (AT) with 333 ms CL stimulation, presented as median (range). F_max_ and A_peak_ were corrected with LMS area.

A_peak_ = peak area. CL = cycle length. CD = contraction duration. CD_50_ = width at 50% of peak height. dF/dt_max_ = steepest positive slope. dF/dt_min_ = steepest negative slope. F_max_ = maximum contraction force. TTP = time to peak. TTR = time to relaxation.
